# Complete vs Incomplete Percutaneous Oblique Distal Closing Wedge Osteotomy for Bunionette (Tailor’s Bunion) Deformity Correction: A Retrospective Comparative Study

**DOI:** 10.1177/24730114261422246

**Published:** 2026-03-04

**Authors:** Sanjana Mehrotra, Ayla Claire Newton, Mohamed Wasim Shaffe Ahamed, Lilanthi Wickramarachchi, Mohamed Sayed Yousef, Peter Lam, Thomas L. Lewis, Robbie Ray

**Affiliations:** 1 School of Medicine and Population Health, Sheffield Medical School, University of Sheffield, United Kingdom; 2 King’s Foot and Ankle Unit, King’s College Hospital NHS Foundation Trust, London, United Kingdom; 3 Orthopaedics and Arthritis Specialist Centre, Sydney, Australia

**Keywords:** bunionette, tailor’s bunion, fifth metatarsal, forefoot deformity, minimally invasive surgery, percutaneous surgery, metatarsal osteotomy, retrospective, outcome studies, callus

## Abstract

**Background:**

There has been increasing interest in the use of percutaneous osteotomy techniques for bunionette (tailor’s bunion) correction. This study evaluated clinical and radiographic outcomes following an unfixed, percutaneous oblique distal metaphyseal-diaphyseal osteotomy and compared outcomes between complete and incomplete osteotomy groups.

**Methods:**

A total of 43 feet (mean age 54.2 ± 17.1) underwent percutaneous oblique distal osteotomy by a single surgeon over a 4-year period. The primary outcome was the presence of significant postoperative hypertrophic callus at the osteotomy site (>150% of the width of the fifth metatarsal shaft), which decreases over time due to normal bone remodeling. Secondary exploratory outcomes included the radiographic parameters fourth-fifth intermetatarsal angle (IMA) and metatarsophalangeal angle (MPA), and patient-reported outcome measures (PROMs) of Manchester-Oxford Foot Questionnaire (MOXFQ), EuroQol 5-Dimension, 5-Level (EQ-5D-5L), and visual analogue scale (VAS) Pain (minimum 12 months’ follow-up).

**Results:**

Thirty feet had a complete osteotomy, and 13 feet had an incomplete osteotomy with the lateral cortex remaining intact. In the complete osteotomy group, 60% of patients (*P* = .001) had callus equivalent to >150% of the metatarsal width at 6-week follow up; this reduced to 19% (*P* = .31) at 6 months and 0% (*P* = 1) at 12 months. No significant hypertrophic callus was observed in the incomplete osteotomy group. All PROMs, except EQ-5D-5L VAS, showed significant improvements (*P* < .05). The IMA and MPA significantly decreased postoperatively across both groups (*P* < .001). There were no significant differences between the incomplete and complete osteotomy groups at follow-up radiographically and clinically, except for the MOXFQ Walking/Standing Domain (*P* = .014), where patients in the incomplete osteotomy group demonstrated greater improvement.

**Conclusion:**

Unfixed, minimally invasive oblique distal osteotomy for bunionette deformity is a safe and effective procedure that is associated with significant improvement in radiographic and clinical outcomes. Whether or not the osteotomy is complete does influence hypertrophic callus formation but does not significantly affect the radiographic or clinical outcomes.

**Level of Evidence:**

Level III, retrospective comparative study.

## Introduction

A bunionette is a lateral deformity of the fifth metatarsal, typically presenting as a painful, erythematous, hyperkeratotic bony prominence on the lateral, plantar, or dorsolateral aspect of the foot. Symptoms of chronic pain and irritation commonly result from friction between footwear and the bony deformity. Surgical intervention is considered when conservative measures, such as wide-toe footwear, padding, metatarsal bars, insoles, or orthoses fail to provide symptom relief.^1-3^

**Figure 1.**
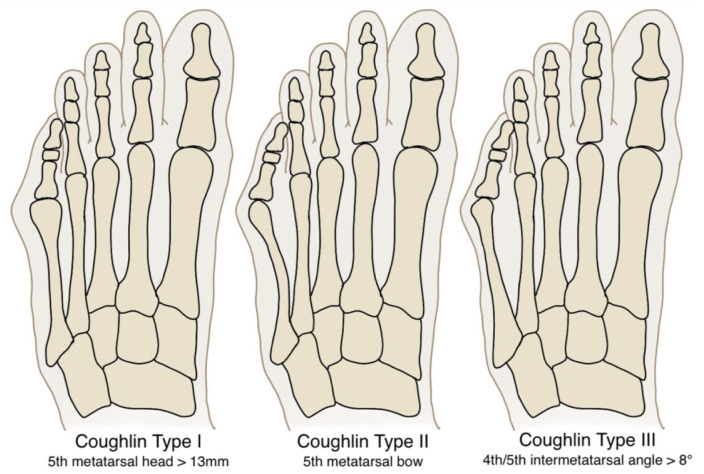
Coughlin’s radiographic classification.

Coughlin et al^4^ classified bunionettes anatomically into 3 types, with type 3, characterised by an increased fourth-fifth intermetatarsal angle (IMA). Type 1 involves an enlarged fifth metatarsal head, whereas type 2 is marked by lateral bowing of the fifth metatarsal shaft (see Figure 1).

De Prado^5^ first introduced the concept of percutaneous, incomplete osteotomy initially, in the correction of hallux valgus. These MIS principles were subsequently adapted for bunionette correction. There is now a range of fixed and unfixed percutaneous osteotomy techniques for bunionette correction. Distal osteotomies have been reported to have better patient-reported outcomes and fewer complications than proximal and diaphyseal osteotomies.^6^ A recent systematic review revealed that hypertrophic callus formation is a commonly observed radiographic finding of unfixed complete osteotomies.^7^

**Figure 2.**
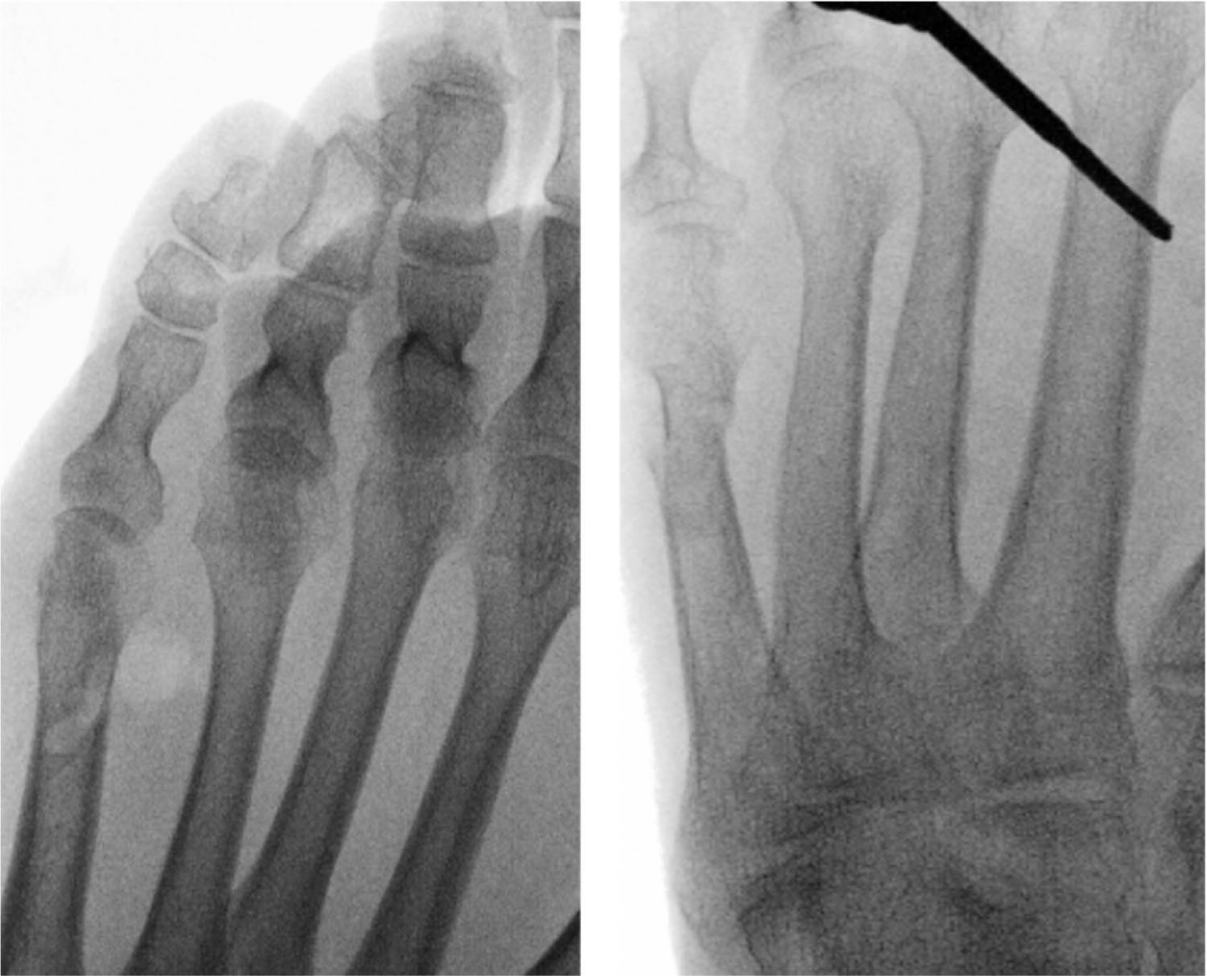
Intraoperative fluoroscopic comparison of bunionette osteotomy techniques. Left image: Intraoperative fluoroscopic image demonstrating an incomplete distal fifth metatarsal osteotomy with an intact lateral cortical hinge, maintaining lateral bone continuity and allowing controlled medial displacement of the metatarsal head. Right image: Intraoperative fluoroscopic image showing a complete osteotomy with loss of the lateral cortical hinge, resulting in full separation of the distal fragment.

Unfixed, incomplete oblique distal osteotomy at the fifth metatarsal neck to create a closing medial wedge, was initially described and taught by De Prado.^8^ This is desirable as it avoids formation of large hypertrophic callus, which may be temporarily symptomatic while it resolves. However, the dorsolateral cortex, serving as a hinge, can fracture intraoperatively, leading to an unstable configuration (see Figure 2). Some studies of minimally invasive Akin osteotomies for hallux valgus report a significant delay in healing in patients with compromise of the lateral cortex, while others find no such significant link.^9-12^

**Figure 3.**
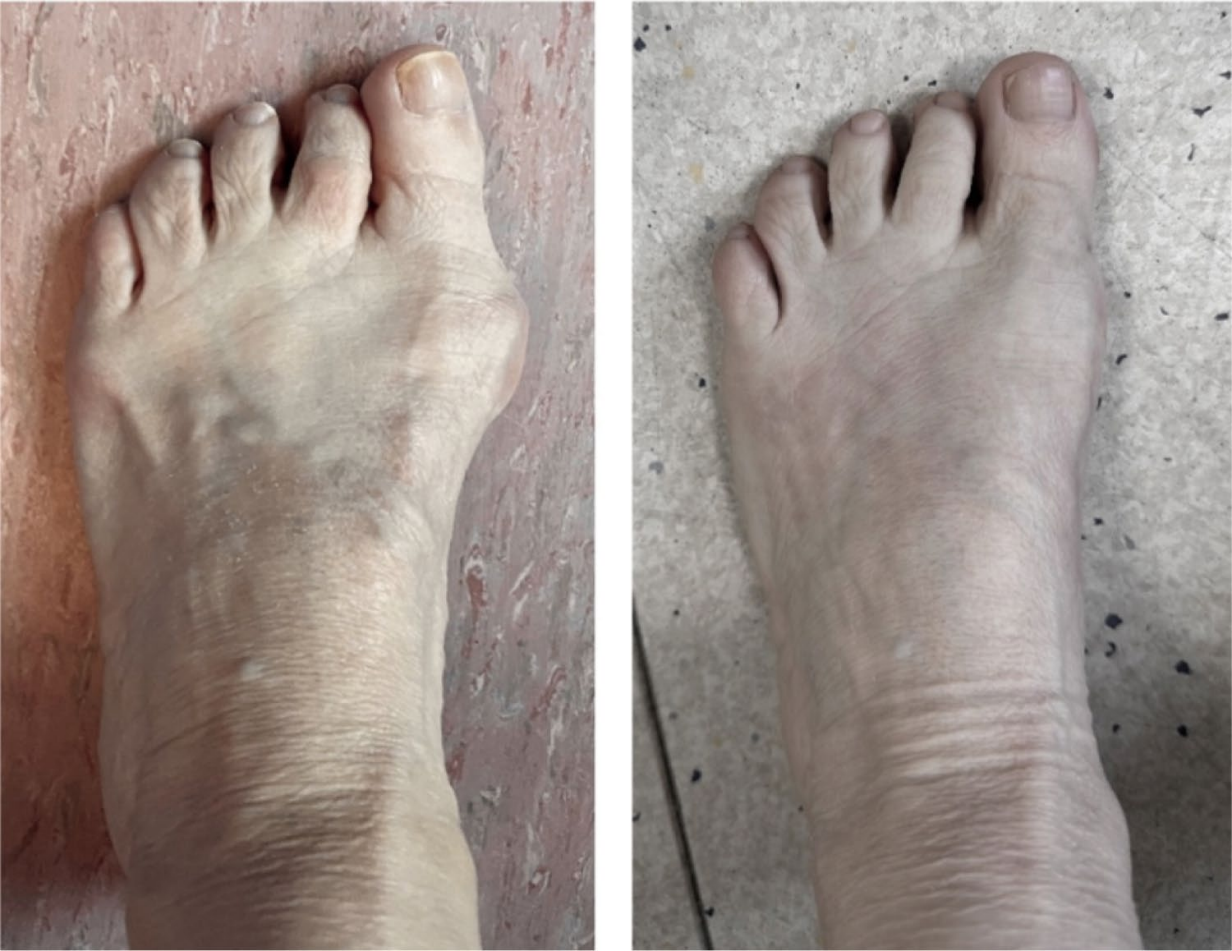
Pre- and post-operative clinical images of the left foot following percutaneous bunionette correction. Left: Pre-operative image showing bunionette and hallux valgus deformity. Right: Six-month postoperative image demonstrating restored alignment and correction.

There remains some debate within the literature regarding the merits of incomplete versus complete osteotomies in percutaneous bunionette correction. Michels et al^13^ noted that while incomplete osteotomies may allow for quicker healing, they can limit the degree of desired metatarsal head translation and therefore carry a risk of undercorrection, particularly in more severe deformities. Similarly, in a commentary, Laffenetre alluded to this ongoing controversy, suggesting that the choice between incomplete and complete techniques continues to be a point of discussion in contemporary practice.^14^ This study is the first to evaluate whether the type of osteotomy performed, specifically, incomplete, preserving the lateral cortex, versus complete osteotomy, affects the size of callus formation and whether there is any significant impact on radiologic or clinical outcomes of that in unfixed, percutaneous oblique distal osteotomy for bunionette correction (see Figure 3).

## Methods

### Operative Technique

**Figure 4.**
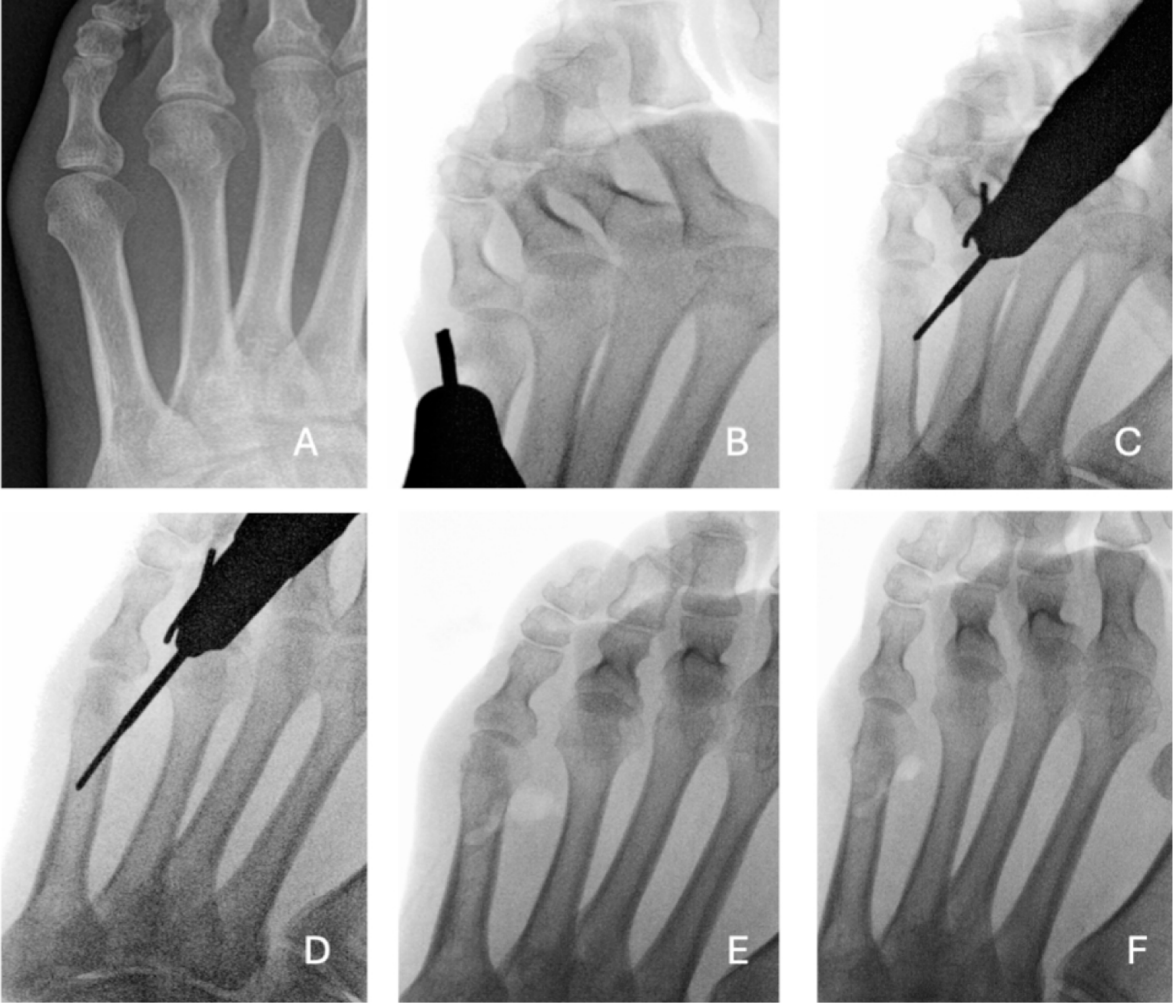
Pre- and intra-operative fluoroscopic sequence of bunionette correction using a Shannon burr. (A) Pre-operative anteroposterior radiograph showing lateral prominence of the fifth metatarsal head consistent with bunionette deformity. (B) Fluoroscopic image showing minimal lateral condylectomy performed using a Shannon burr. (C) Establishing the entry point for the osteotomy just proximal to the metatarsal head using the same burr. (D) The burr is advanced in a controlled manner to complete the osteotomy by cutting through both the dorsal and plantar cortices. (E) Completion of the osteotomy; the distal fragment is manually manipulated to achieve desired correction and closure. (F) Final fluoroscopic image showing the corrected alignment and final position of the fifth metatarsal following osteotomy and manual reduction.

The patient is positioned supine under appropriate anaesthesia (regional ankle block or general anesthesia). A thigh tourniquet is applied as part of our standard operative setup to improve visualisation and is inflated to 300 mm Hg. A 2- mm longitudinal incision is made just medial to the head of the fifth metatarsal. A periosteal elevator is then used to carefully dissect and retract the extensor tendon, ensuring its protection throughout the procedure. Any prominent lateral condyle of the fifth metatarsal head can be addressed using 2 × 12-mm Shannon burr mounted on a low-speed, high-torque power system to perform a minimal lateral condylectomy in all cases to reduce residual bony prominence. Care is taken to avoid excessive bone resection, which could lead to instability of the fifth metatarsophalangeal joint. The osteotomy is a closing wedge oblique distal incomplete osteotomy oriented from distal-medial to proximal-lateral. The starting point is at the central lateral aspect of the fifth metatarsal neck, approximately 1 cm proximal to the metatarsal head. The osteotomy is initiated via an entry portal created dorsomedially in the fourth-fifth intermetatarsal webspace, with the Shannon burr directed at a 45-degree angle to the long axis of the metatarsal, and its position confirmed using fluoroscopy. The medial, dorsal, and plantar cortices are cut, leaving the dorsolateral cortex intact to serve as a hinge (see Figure 4). During the cut, manual pressure is applied to the lateral aspect of the fifth metatarsal head. When displacement is felt, the cut is stopped to preserve the dorsolateral hinge, while closing the wedge and reducing the intermetatarsal angle. The fifth ray is gently manipulated to achieve the desired correction of the bunionette deformity, with the metatarsal head being shifted medially and confirmed with fluoroscopic imaging. There is no internal fixation used, and no osteotomy of the P1 of the fifth toe is used. The incision is irrigated with normal saline. The skin is closed with a single absorbable 4-0 braided suture. Patients are allowed to weight-bear as tolerated in a postoperative stiff-soled shoe for 6 weeks. Active and passive range-of-motion exercises of the fifth toe are encouraged starting at 2 weeks postoperatively. Taping is used to maintain the position of the toe for 6 weeks.

### Study Design

This study is a retrospective analysis of prospective data from consecutive patients undergoing unfixed, percutaneous oblique distal osteotomy for bunionette correction. The study was reported in line with the STROBE guidelines for observational studies.^15^

### Study Setting

Participants were recruited across a 4-year timeframe, from February 2020 to January 2024. The same technique was performed by a single consultant surgeon who is trained in percutaneous techniques of foot and ankle surgery. All procedures were performed in 2 hospitals in London, United Kingdom.

### Participants

All patients aged ≥18 years with persistent symptoms despite at least 6 months of conservative management, including orthoses, analgesia, and activity modification, were considered for surgery. Inclusion criteria comprised patients diagnosed with a bunionette according to Coughlin’s classification who were eligible for an unfixed, percutaneous oblique distal fifth metatarsal osteotomy.^2^ All radiologic severities were included. Patients undergoing additional forefoot or calf procedures, including lesser toe corrections, PIPJ arthrodesis, Weil osteotomies, or proximal medial gastrocnemius release, were included. Revision procedures were excluded.

### Group Allocation

The intent was to perform an incomplete medial closing wedge oblique osteotomy of the distal diaphyseal-metaphyseal junction of the fifth metatarsal. In a subset of patients, the osteotomy was completed unintentionally, either at the time of using the burr to make the osteotomy or when performing the reduction maneuver of squeezing the metatarsals. It should be noted that incomplete osteotomies may be inherently limited in the degree of correction achievable. In cases of larger deformities, achieving the desired metatarsal head translation may have fractured the lateral cortex, unintentionally converting the osteotomy to a complete cut. These patients therefore had complete osteotomies, and we compared their outcomes to those who had the intended incomplete osteotomy. This resulted in patients being allocated to either the incomplete or complete group after the procedure.

### Data Sources/Collection

Each patient prospectively completed pre-operative patient-reported outcome measures (PROMs), which were stored in a national online registry on the day of the surgery. Patients completed follow-up PROMs at routine time points postoperatively. Radiologic assessment was performed at 6 weeks, 6 months, and 1 year.

### Variable, Outcome Measures, and Study Endpoint

General demographic details and additional procedures were obtained from clinical records.

The primary outcome was the presence of significant hypertrophic callus at the osteotomy site on the post-operative radiographs, which we defined as >150% of the width of the fifth metatarsal shaft at the level of the osteotomy on the dorsal-plantar radiograph. This was chosen based on the assumption that >150% may be large enough to be symptomatic for patients. This outcome was selected because hypertrophic callus formation has been reported as a common and potentially symptomatic finding following unfixed percutaneous complete osteotomies,^14^ and to assess whether preservation of the lateral cortical hinge influences radiologic or clinical findings.

Secondary outcomes included radiologic and clinical parameters. The two radiologic outcomes were the fourth-fifth intermetatarsal angle (IMA) and fifth metatarsophalangeal angle (MPA). Clinical outcomes comprised validated PROMs, including the Manchester-Oxford Foot Questionnaire (MOXFQ), EuroQol 5-Dimension, 5-Level (EQ-5D-5L) visual analogue scale (EQ-VAS), and pain visual analogue scale (VAS-Pain) assessed at a minimum of 12 months following surgery.

Complications identified by either the patient or surgeon in outpatient clinics were prospectively recorded at the point of diagnosis and categorised using the Adapted Clavien-Dindo complication classification.^16^

### Bias

Potential sources of bias were addressed within the study design. The operating surgeon was not involved in data analyses. Radiographic measurements of IMA and MPA were performed by independent observers blinded to PROM outcomes. Consecutive patients were included with minimal exclusion criteria to reduce selection bias and maximise sample size. Prospective data collection was used to minimise recall bias.

### Statistical Analysis

Paired Student *t* tests were used for parametric continuous outcomes and Fisher exact tests for categorical data. Analyses were performed using RStudio. A subgroup analysis was conducted for isolated bunionette procedures. Statistical significance was set at *P* <.05.

### Ethical Approval and Funding

No external funding was obtained for this study. This study was registered as a local service evaluation using routinely collected data, including prospectively gathered PROMs.

## Results

### Patient Demographics

Forty-three feet from 34 patients were operated on between February 2020 and January 2024. Of these, 30 feet underwent a complete osteotomy, and 13 feet had an incomplete osteotomy, with the lateral cortex remaining intact. Twenty-eight patients were female (82%), and 6 were male (18%). The mean age at the time of operation was 54.2 ± 17.1 years. Body mass index mean was 25.1 ± 4.0. Nineteen left feet (44%) and 24 right feet (56%) were operated on. Twelve cases (28%) had isolated bunionette correction. Thirty cases (70%) had additional hallux valgus correction of the same foot. Twelve cases (28%) had other lesser toe procedures including osteotomies and arthrodesis (Table 1).

**Table 1.** Patient Demographics.

Characteristic	Incomplete Osteotomy (n = 13)	Completed Osteotomy (n = 30)	All Cases (N = 43)
Gender			
Male	4	3	7
Female	9	27	36
Age, mean ± SD	40.0 ± 15.4	60.4 ± 14.0	54.2 ± 17.1
BMI, mean ± SD	26.8 ± 3.9	24.3 ± 3.9	25.1 ± 4.0
Side			
Left	6	13	19
Right	7	17	24
Other procedures			
Isolated bunionette	6	6	12
HV correction ± lesser toe procedures	7	23	30
Other lesser toe procedures	0	1	1

Abbreviations: BMI, body mass index; HV, hallux valgus.

### Primary Outcome

Post-operative radiographs were available for 42 cases (98%) (see Table 2). The primary clinical outcome was presence of significant hypertrophic callus at the osteotomy site on the post-operative radiographs which we defined as being >150% of the width of the fifth metatarsal shaft at the level of the osteotomy on the dorsal-plantar radiograph. Post-operative radiographs for at least one timepoint were available for 42 out of 43 patients. Fisher tests were performed at each time point to see if the number of cases that had >150% of the width of the metatarsal was significantly different between the complete and incomplete osteotomy groups. Statistical significance was determined only at the 6 week mark (*P* = .0003) and not at the 6-month (*P* = .31) or 12-month mark (*P* = 1).

**Table 2.** Radiographic Outcome Analysis Stratified by Completion of Osteotomy of Fifth Metatarsal.

Time point	Pre-operative	6 wk	6 mo	12 mo
Incomplete osteotomy (n = 13)				
Number at time point, n (%)	13	12 (92.3)	5 (38.4)	5 (38.4)
Time post-surgery, mean ± SD	-	8.2 ± 3.7 wk	5.6 ± 0.9 mo	14.6 ± 4.3 mo
Coughlin classification, n (%)		-	-	-
Type 1	7 (54)			
Type 2	2 (15)			
Type 3	4 (31)			
Fourth-fifth IMA, degrees, mean ± SD	9.0 ± 1.9	5.4 ± 1.2	4.8 ± 1.4	5.5 ± 1.0
Fifth MPA, degrees, mean ± SD	12.5 ± 4.9	7.2 ± 2.9	5.0 ± 3.2	5.9 ± 3.3
Callus >150%, n (%)	-	0 (0)	0 (0)	0 (0)
Completed osteotomy (n = 30)				
Number at time point, n (%)	30	30 (100)	25 (83)	18 (60)
Time post-surgery, mean ± SD	-	7.4 ± 2.6 wk	5.5 ± 2.3 mo	14.0 ± 3.7 mo
Coughlin classification, n (%)		-	-	-
Type 1	9 (30)			
Type 2	6 (20)			
Type 3	15 (50)			
Fourth-fifth IMA, degrees, mean ± SD	10.5 ± 2.7	6.4 ± 2.3	5.2 ± 2.4	5.0 ± 1.9
Fifth MPA, degrees, mean ± SD	14.4 ± 5.5	5.6 ± 3.5	4.7 ± 2.9	4.6 ± 2.5
Callus >150%, n (%)		18 (60)**	5 (19)	0 (0)

Abbreviations: IMA, intermetatarsal angle; MPA, metatarsophalangeal angle.

**P < .01.

### Secondary Outcomes

Secondary outcomes were post-operative radiographic deformity correction of the fourth-fifth inter-metatarsal angle (IMA) and the fifth metatarsophalangeal angle (MPA) (Table 2) and PROMs of MOXFQ, EQ-5D-5L, and VAS Pain (Table 3). Overall, across both groups, there was a statistically significant improvement in the IMA from a mean of 10.0 ± 2.6 pre-operatively to 5.4 ± 2.0 post-operatively (*P* < .001) and MPA from a mean of 13.8 ± 5.3 to 5.4 ± 3.0 post-operatively (*P* < .001). There was a statistically significant difference between the incomplete and complete osteotomy groups pre-operative fourth-fifth inter-metatarsal angle (IMA) (*P* = .04) but not for the post-operative IMA (*P* = .66). There was no statistically significant difference between the incomplete and complete osteotomy groups fifth metatarsophalangeal angle (MPA) pre-operatively (*P* = .28) or post-operatively (*P* = .41).

**Table 3.** Patient-Reported Outcome Measures Stratified by Completion of Osteotomy of fifth Metatarsal.

Outcome Measure	Pre-operative, Mean ± SD	Post-operative, Mean ± SD	Improvement, Mean (95% CI)	Partial vs Completed *P* Value
Incomplete osteotomy (n = 13)				
MOXFQ				
Pain	47.3 ± 20.4	9.2 ± 16.1	38.1 (24.7 to 51.4) **	.06
Walking/Standing	51.9 ± 21.5	7.0 ± 11.2	44.9 (31.2 to 58.7) **	.014*
Social Interaction	44.8 ± 23.6	8.6 ± 19.8	36.2 (22.1 to 50.4) **	.22
EQ-5D-5L				
VAS Pain	37.3 ± 24.8	11.5 ± 21.9	25.8 (8.9 to 42.7) **	.36
EQ-VAS	83.5 ± 14.4	86.5 ± 11.3	3.0 (−8.5 to 14.5)	.56
Index	0.72 ± 0.13	0.91 ± 0.19	0.2 (0.1 to 0.3) *	.12
Completed osteotomy (n = 28)				
MOXFQ				
Pain	41.8 ± 16.7	20.2 ± 19.8	21.6 (12.1 to 31.2) **	.06
Walking/Standing	34.5 ± 25.4	13.3 ± 16.3	21.2 (10.0 to 32.4) **	.014*
Social Interaction	38.9 ± 22.6	14.2 ± 15.3	24.7 (13.7 to 35.7) **	.22
EQ-5D-5L				
VAS Pain	34.2 ± 26.8	18.6 ± 21.5	15.6 (2.2 to 29.0) *	.36
EQ-VAS	74.9 ± 15.3	73.3 ± 23.2	−1.6 (−11.7 to 8.5)	.56
Index	0.74 ± 0.16	0.78 ± 0.20	0.1 (0.0 to 0.1)	.12

Abbreviations: EQ-5D-5L, EuroQol 5-Dimension, 5-Level; MOXFQ, Manchester-Oxford Foot Questionnaire; VAS, visual analogue scale.

*P < .05, **P < .01.

**Figure 5.**
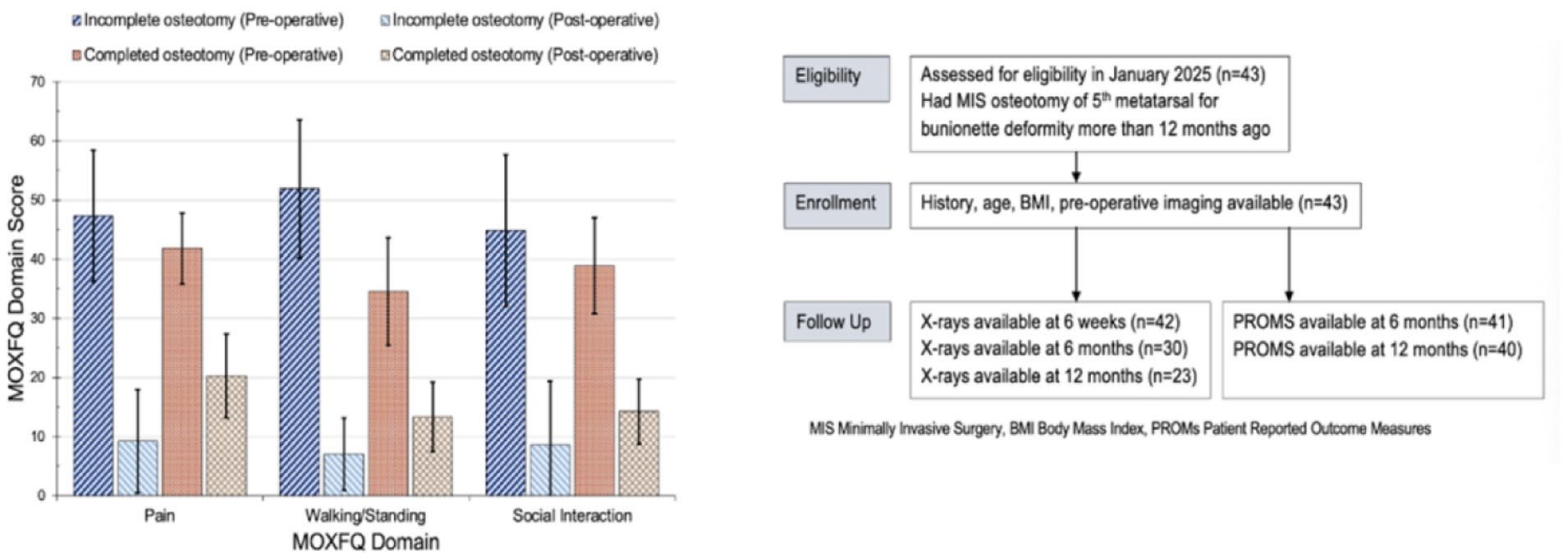
The left chart demonstrates improvement in MOXFQ domains following bunionette correction in both groups, and on the right, the participant flow diagram demonstrates eligibility, enrollment, and follow-up.

PROMs were available for 41 cases (95%) at a mean follow-up of 21.7 ± 9.3 months, with 40 cases (93%) having completed at least 12 months after surgery. Figure 5 summarises enrolment and follow-up. Overall across both groups, the MOXFQ Pain score improved from a mean of 43.5 ± 17.9 pre-operatively to 16.7 ± 19.2 post-operatively (*P* = .003), the MOXFQ Social Interaction score improved from a mean of 39.2 ± 24.5 pre-operatively to 16.2 ± 24.7 post-operatively (*P* = .002), and the MOXFQ Walking score improved from a mean of 38.1 ± 27.2 pre-operatively to 14.3 ± 21.4 post-operatively (*P* = .004). Table 3 demonstrates a statistically significant improvement in all MOXFQ domains following surgery stratified according to whether the osteotomy was completed or not. Other secondary outcomes included the EQ-5D-5L Index and VAS Pain, both of which also showed statistically significant improvement across both groups from pre-operative to post-operative (*P* = .0005 and *P* = .001, respectively), and EQ-5D-5L VAS, which did not show statistically significant improvement (*P* = .97). Table 3 shows these scores stratified according to whether the osteotomy was completed or not. This analysis included patients who underwent isolated bunionette correction as well as those who had additional procedures. There was no significant difference between the improvement in any of the PROMs between the incomplete and complete osteotomy group apart from the MOXFQ Walking/Standing Domain (*P* = .014). We did not have PROMs at the early stages of healing to see if there was a difference between the 2 groups in terms of early healing.

The 12 cases that underwent isolated bunionette correction had an improvement across all MOXFQ Domains: Pain, 30.0 ± 20.2 pre-operatively to 3.1 ± 7.2 post-operatively (*P* = .0004); Walking, 30.5 ± 22.8 pre-operatively to 4.5 ± 7.5 post-operatively (*P* = .001); and Social Interaction, 34.3 ± 19.7 pre-operatively to 5.3 ± 9.2 post-operatively (*P* = .05). We did not do further subgroup analysis of complete vs incomplete because of small sample sizes and risk of type 2 error.

### Complications

No complications or wound issues occurred around the fifth metatarsal osteotomy site. There were no incidences of nonunion at the 12-month follow-up point.

## Discussion

**Figure 6.**
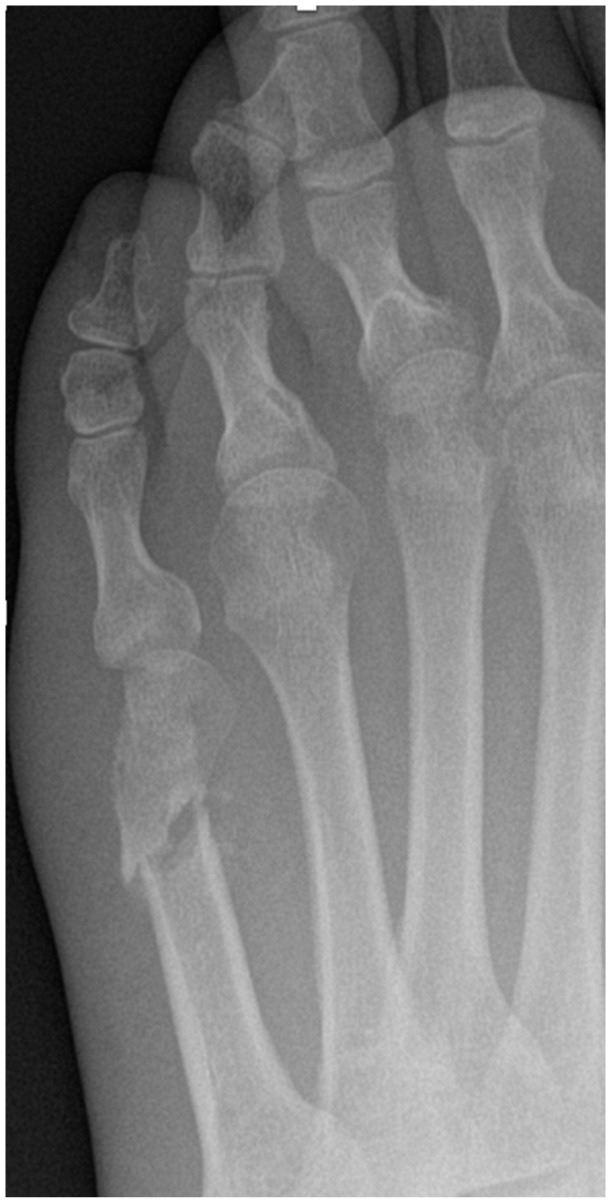
Radiograph showing complete osteotomy with disrupted lateral cortical hinge.

Despite aiming for an incomplete osteotomy, a fracture in the lateral cortex can occur, resulting in a complete osteotomy (Figure 6). An undesirable fracture of the lateral cortex can lead to angulation of the distal fragment and malunion or recurrence. This study found that unfixed, percutaneous oblique distal osteotomy is associated with significant improvement in radiologic and patient-reported outcomes regardless of whether the osteotomy is complete or not. Previous studies have demonstrated positive outcomes when complete and incomplete osteotomies are assessed independently; however, this study is the first to directly compare the 2 techniques and evaluate for clinically and radiographically significant differences between complete and incomplete osteotomy groups.^7,17-19^

**Figure 7.**
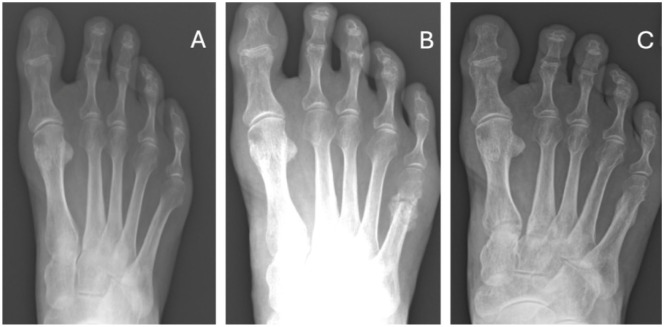
(A) Pre-operative radiograph showing lateral deviation of the fifth metatarsal head. (B) 12-week postoperative radiograph demonstrating osteotomy site healing with significant hypertrophic callus formation. (C) 12-month radiograph showing complete remodeling and resolution of callus at the osteotomy site.

**Figure 8.**
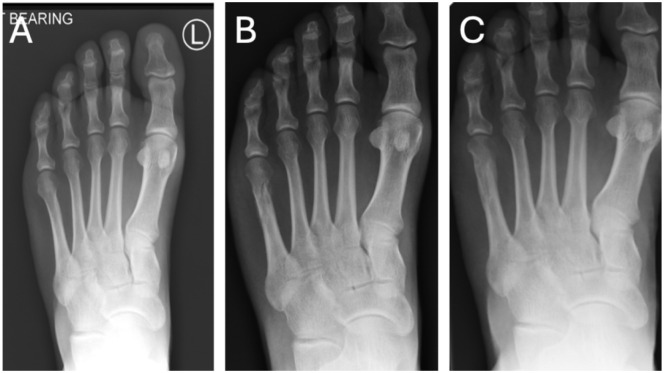
Example of incomplete osteotomy that healed without significant hypertrophic callus formation: (A) pre-operative, (B) 6 weeks post-operative, and (C) 6 months post-operative.

Based on our results, radiologically, there was only significant hypertrophic callus formation at 6 weeks postoperatively (*P* < .01), compared with the 6-month and 12-month time points for the complete osteotomy group (see Figure 7). There was no significant hypertrophic callus formation at any time point for the incomplete osteotomy group (see Figure 8); the reason for this could be improved mechanical stabilisation of the osteotomy provided by the closing medial wedge. Although it has been observed that lack of fixation is an important predictor in callus formation regardless of the type of osteotomy performed,^7,17,19,20^ this study reports that significant hypertrophic callus was only formed in the complete osteotomy group. It would be interesting to identify whether this causes more pain, especially as secondary fracture occurs during the healing process. This could determine the superiority of the “ideal” incomplete cut, whose postoperative effects are minimal, compared with those of complete osteotomies. It is also important to note that operating on the hallux can also be a factor in analgesic supination, which may lead to secondary fracture of the hinge. This finding is supported by a recent systematic review and several studies that report the common formation of a hypertrophic callus post-surgery in unfixed complete osteotomy, which usually self-resolves without intervention.^7^ The lack of fixation and subsequent instability, along with early weight-bearing, may contribute to callus development.^21-24^

Our results showed that IMA and MPA reduced significantly for both the complete and incomplete osteotomy groups, with no significant difference between them (IMA, *P* = .66; MPA, *P* = .41). Despite an increased pre-operative IMA angle (*P* = .04) in patients who had complete osteotomy performed, there was no statistically significant difference postoperatively between the incomplete and the complete osteotomy group. The finding of a significantly higher pre-operative fourth–fifth IMA in the complete osteotomy group suggests that increasing deformity severity may necessitate greater translation for correction, thereby increasing the risk of lateral cortical disruption and limiting the practicality of an incomplete osteotomy in such cases. In more severe deformities, the inherent limitations of angular or rotational correction achievable with an incomplete osteotomy may predispose to intraoperative completion of the osteotomy.

This is the first study to look at comparative outcomes for bunionette corrections between the complete and incomplete osteotomies, assessing the impact of hypertrophic callus formation on outcome measures. We found no significant difference in patient-reported outcomes between groups, with significant improvements in both cohorts in MOXFQ, EQ-5D-5L index, and VAS Pain scores after surgery. A subgroup analysis for 12 cases who only had the bunionette procedure performed revealed significant improvements in MOXFQ scores in all domains.

### Strengths

This is a technical description and clinical evaluation of an established technique, showing its safety and reliability in both isolation and in pragmatic situations associated with other forefoot procedures. Minimal loss to follow-up for PROMs at 12 months post-surgery strengthens the reliability of patient-reported outcomes.

### Limitations

The main limitation is the small subgroup of isolated bunionette corrections and the effect of confounding from other additional procedures performed alongside bunionette corrections. The small number of isolated bunionette corrections limited the ability to perform meaningful comparative analysis between the groups; therefore, findings of no significant difference should be interpreted with caution. Early PROMs were not available, limiting evaluation of pain during the early stages of healing, particularly in complete osteotomies where there is disruption of the lateral cortex. The use of MOXFQ may limit the sensitivity in detecting reliable clinical outcomes because, although validated for foot and ankle surgery, it lacks specificity for bunionette correction. Because of the small subgroup size, analysis of MOXFQ outcomes in these cases should be considered exploratory. As this study is the first of its kind, we could not perform a priori sample size calculation and therefore included as many patients as possible. Additionally, lack of randomisation and single surgeon technique affect the generalisability.

## Conclusion

Unfixed, percutaneous oblique distal metaphyseal-diaphyseal osteotomy for bunionette correction is associated with significant improvement in clinical and radiologic outcomes regardless of whether the osteotomy is complete or incomplete. It was noted that a greater deformity correction may inevitably lead to a complete osteotomy during correction maneuvers. Complete osteotomies frequently lead to significant hypertrophic callus formation in the early stages of healing, possibly due to the secondary fracture of the hinge. Future studies could explore whether this is associated with differences in early postoperative pain. Callus resolved spontaneously without surgical intervention. Further studies with larger sample sizes undergoing bunionette correction as an isolated procedure are needed to assess this finding with greater reliability.

## Supplemental Material

sj-pdf-1-fao-10.1177_24730114261422246 – Supplemental material for Complete vs Incomplete Percutaneous Oblique Distal Closing Wedge Osteotomy for Bunionette (Tailor’s Bunion) Deformity Correction: A Retrospective Comparative Study Supplemental material, sj-pdf-1-fao-10.1177_24730114261422246 for Complete vs Incomplete Percutaneous Oblique Distal Closing Wedge Osteotomy for Bunionette (Tailor’s Bunion) Deformity Correction: A Retrospective Comparative Study by Sanjana Mehrotra, Ayla Claire Newton, Mohamed Wasim Shaffe Ahamed, Lilanthi Wickramarachchi, Mohamed Sayed Yousef, Peter Lam, Thomas L. Lewis and Robbie Ray in Foot & Ankle Orthopaedics
